# Editorial: Radiomics and AI for clinical and translational medicine

**DOI:** 10.3389/fradi.2024.1375443

**Published:** 2024-02-19

**Authors:** Eros Montin, Valentina D. A. Corino, Dimitri Martel, Giuseppe Carlucci, Davide Scaramuzza

**Affiliations:** ^1^ Department of Radiology, Bernard and Irene Schwartz Center for Biomedical Imaging, New York University Grossman School of Medicine, New York, NY, United States; ^2^ Department of Radiology, Center for Advanced Imaging Innovation and Research (CAI2R), New York University Grossman School of Medicine, New York, NY, United States; ^3^ Dipartimento di Elettronica, Informazione e Bioingegneria, Politecnico di Milano, Milan, Italy; ^4^ CardioTech Lab, IRCCS Centro Cardiologico Monzino, Milan, Italy; ^5^ Biomedical Cyclotron Facility Ahmanson Translational Theranostics Division, University of California, Los Angeles, Los Angeles, CA, United States; ^6^ Dipartimento di Diagnostica per immagini e radioterapia, Fondazione IRCCS Istituto dei Tumori, Milan, Italy

**Keywords:** radiomics, genomics, artificial intelligence, features selection (FS), machine learning, automatic diagnosis and prediction models

**Editorial on the Research Topic** Radiomics and AI for clinical and translational medicine

In the past decade, a revolution has quietly blossomed in the realm of medical diagnostics. Radiomics, the extraction of quantitative features from medical images like CT, PET, and MRI, has emerged as a powerful tool for uncovering hidden patterns invisible to the naked eye. These patterns hold the key to unlocking groundbreaking insights into disease diagnosis, treatment prediction, and ultimately, improved patient care.

This Research Topic, titled *Radiomics and AI for clinical and translational medicine,* shines a spotlight on six cutting-edge articles exploring the latest advancements in this captivating field. Each study delves into specific medical challenges, showcasing how radiomics, often in partnership with artificial intelligence, can revolutionize diagnosis and treatment across diverse specialties.

One such example is the review article by Shu et al., which delves into the potential of radiomics for managing immune checkpoint inhibitor-related pneumonitis (ICIP) in non-small cell lung cancer (NSCLC) patients. This debilitating side effect can significantly impact treatment, and early detection is crucial. The authors meticulously dissect the underlying mechanisms of ICIP, the radiomics feature extraction process, and its applications in diagnosis, differentiation, and prediction. Their well-structured review, supported by robust evidence and a balanced perspective, paints a promising picture for radiomics in tackling this challenging complication.

Cancer immunotherapy has undoubtedly transformed cancer treatment, but predicting who will respond remains a significant hurdle. The study by Bouhamama et al. shines a light on how radiomics and genetic analyses can be combined to identify potential biomarkers for response to PD-L1 inhibitor therapy in NSCLC patients. By building models based on both tumor imaging features (radiomics) and gene expression data (transcriptomics), they achieved an impressive prediction accuracy exceeding 90% in the testing set. This paves the way for personalized immunotherapy regimens, tailoring treatment to individual patients for optimal outcomes.

Accurate and swift diagnosis of kidney cancers is crucial for optimal patient care. Zhai et al. tackle the challenge of differentiating between infiltrative renal cell carcinoma (RCC) and pyelocaliceal upper urinary tract urothelial carcinoma (UTUC), two deceptively similar tumors. Employing radiomics features extracted from CT scans, they built a model with a remarkable 90% accuracy in the testing set. This translates to fewer misdiagnoses, earlier treatment, and ultimately, improved patient outcomes. The impact is potentially immense, enabling surgeons to plan surgeries effectively, avoiding unnecessary biopsies, and improving patient recovery times.

Moving beyond cancer, the study by Martel et al. explores the potential of radiomics in tackling osteoporosis, a condition that weakens bones and increases fracture risk. They demonstrate how models trained using MRI-based radiomics features can automate osteoporosis assessment, potentially paving the way for earlier intervention and personalized treatment plans. The accuracy of their model, exceeding 0.7 in the area under the curve (AUC), suggests exciting possibilities for preventing fractures and improving patient outcomes.

Distinguishing cardiac amyloidosis (CA) from aortic stenosis (AS) is another daunting challenge for cardiologists, as both masquerades with similar symptoms. However, mistaking one for the other can have dire consequences due to their vastly different prognoses. Lo Iacono et al. present a promising radiomics-based approach for diagnosing these types of tumors. Their classification model, built using radiomics features extracted from CT scans, boasts a 93% success rate in differentiating CA from AS. This breakthrough has the potential to save lives and guide optimal treatment by simplifying the diagnostic process and empowering physicians to make informed decisions.

Finally, the study by Montin et al. tackles the tricky diagnosis of femoroacetabular impingement (FAI), a painful hip condition caused by bone misalignment. Traditionally, diagnosis relies on subjective and time-consuming manual analysis of MRI images. However, Montin et al. open the doors to a game-changer: radiomics-based automatic FAI detection. By analyzing MRI images of patients with symptomatic and asymptomatic FAI, they identified a set of radiomics features that effectively distinguish between the two groups. Their most promising model achieved an astounding accuracy exceeding 97%, surpassing even the 90% accuracy of traditional methods. This suggests that radiomics could revolutionize FAI diagnosis by offering a faster, more objective, and potentially more accurate method, ultimately improving patient care and management of this painful condition.

In conclusion, this Research Topic paints a vivid picture of the transformative power of radiomics. From accurately diagnosing complex tumors to predicting treatment response and automating tedious tasks, radiomics is emerging as a powerful tool for personalized, data-driven healthcare. While further research and validation are necessary for seamless clinical integration, the presented evidence undeniably suggests a future where radiomics empowers clinicians with the tools for earlier diagnoses, personalized treatment plans, and improved patient outcomes. As this dynamic field continues to evolve, we can expect even more groundbreaking discoveries, ultimately revolutionizing the way we diagnose and treat diseases, leading to a future where healthcare is tailored to the individual, maximizing the chances of successful treatment and minimizing unnecessary interventions.

